# Draft Genome Sequence of Multidrug-Resistant Proteus mirabilis CKTH01, Isolated from Raw Chicken Meat

**DOI:** 10.1128/MRA.00861-19

**Published:** 2019-09-19

**Authors:** Supatra Areekit, Nuttida Thongpramul, Wariya Yamprayoonswat, Watthanachai Jumpathong, Satapanawat Sittihan, Suwanan Wanthongchareon, Pattarawan Ruangsuj, Sirirat Wachiralurpan, Kosum Chansiri, Montri Yasawong

**Affiliations:** a Innovative Learning Center, Srinakharinwirot University, Bangkok, Thailand; b Center of Excellence in Biosensors, Srinakharinwirot University, Bangkok, Thailand; c Environmental Toxicology, Chulabhorn Graduate Institute, Chulabhorn Royal Academy, Bangkok, Thailand; d Center of Excellence on Environmental Health and Toxicology (EHT), Office of Higher Education, Bangkok, Thailand; e Chemical Biology, Chulabhorn Graduate Institute, Chulabhorn Royal Academy, Bangkok, Thailand; f Maintenance Technology Center, Institute for Scientific and Technological Research and Services, King Mongkut’s University of Technology Thonburi, Bangkok, Thailand; g Department of Biochemistry, Faculty of Medicine, Srinakharinwirot University, Bangkok, Thailand; Loyola University Chicago

## Abstract

Proteus mirabilis CKTH01 is a pathogenic bacterium isolated from raw chicken meat. The genome sequence of P. mirabilis CKTH01 contains genes encoding multidrug efflux pumps, which are the virulence factors of the antibiotic-resistant bacterium. This 3.98-Mb draft genome sequence of P. mirabilis CKTH01 will contribute to the understanding of the distribution of multidrug-resistant P. mirabilis in raw chicken meat at the open markets.

## ANNOUNCEMENT

Proteus mirabilis is a facultatively anaerobic, Gram-negative bacterium (1, 2). The optimum growth temperature of P. mirabilis is 37°C, and it can swarm on solid medium using peritrichous flagella (2). It is a major cause of catheter-associated urinary tract infections (CAUTI), especially in patients who have long-term catheterization (3).

Bacterial strains were isolated from raw chicken meat samples from open markets in Bangkok, Thailand. Raw chicken meat (10 g) was put into a stomacher bag containing 90 ml peptone water and then homogenized using a stomacher. The mixture (100 μl) was spread on xylose-lysine-deoxycholate (XLD) agar and incubated overnight at 37°C. A single colony of bacterial strain CKTH01 was grown in tryptic soy broth at 37°C with shaking at 250 rpm for 16 h. Genomic DNA of strain CKTH01 was extracted by the phenol-chloroform method described by Sambrook and Russell (4). A NanoDrop spectrophotometer (Thermo Fisher Scientific, USA) was used to determine the quality and quantity of genomic DNA. A sequencing library was prepared using the Ion PI Hi-Q OT2 template and Ion Plus fragment library kits. The library was placed on an Ion PI chip, and sequencing was performed using an Ion Proton sequencer (Thermo Fisher Scientific). The average read length was 110 bp. There were 8,117,310 raw reads (223× coverage depth) generated from the sequencing run. The draft genome sequence of strain CKTH01 was identified using JSpecies Web server (JSpeciesWS) (5). Bacterial strain CKTH01 was affiliated with Proteus mirabilis with 98.97% supported value of the average nucleotide identity (ANI) based on BLAST+ (ANIb). The difference in G+C content between strain CKTH01 and the type strain of P. mirabilis was 0.29%.

The quality of the raw reads was determined using AfterQC version 0.9.6 with default parameters (6). *De novo* genome assembly was performed with the raw reads and SPAdes version 3.13.1 in “–careful” mode (7). The genome assembly metric was determined using QUAST version 5.0.2, with default parameters (8). The draft genome sequence of P. mirabilis CKTH01 consists of a single chromosome (3,976,359 bp in 590 contigs), with an *N*_50_ value of 20,445 bp and a G+C content of 38.81%. Gene prediction was performed using Prokka version 1.13.7, with default parameters (9). The annotated genome sequence of P. mirabilis CKTH01 contains 3,774 total genes, 3,703 protein-coding sequences, 65 tRNA genes, 5 rRNA genes, and 1 transfer-messenger RNA (tmRNA) gene.

There were 28 annotated loci in the P. mirabilis CKTH01 genome that encoded multidrug efflux pumps in different families, including the ATP-binding cassette (ABC) family, the major facilitator superfamily (MFS), the multidrug and toxin extrusion (MATE) family, and the resistance-nodulation-cell division (RND) superfamily.

These results suggest that P. mirabilis CKTH01 is a multidrug-resistant bacterium. The genome of P. mirabilis CKTH01 will facilitate an understanding of the distribution of multidrug-resistant P. mirabilis in raw chicken meat at the open markets.

### Data availability.

The whole-genome shotgun sequence of Proteus mirabilis CKTH01 has been deposited at DDBJ/ENA/GenBank under accession number VHPV00000000 and in the SRA under accession number SRR9587835.
